# Compressive Strength and CO_2_ Mineralization Mechanism of Copper Slag-GGBS Alkali-Activated Geopolymer Composites Enhanced by MgO and Biochar

**DOI:** 10.3390/ma18194434

**Published:** 2025-09-23

**Authors:** Quanbin Jin, Wei Xiang, Chenghua Xu, Guoyi Tang, Zhibin Liu

**Affiliations:** 1 Institute of Geotechnical Engineering, Southeast University, Nanjing 211189, China; seulzb@seu.edu.cn; 2 Jiangsu Key Laboratory of Low Carbon and Sustainable Geotechnical Engineering, Southeast University, Nanjing 211189, China; 3 Shenzhen Road and Bridge Construction Group Co., Ltd., Shenzhen 518024, China; xiangwei0088@126.com; 4 Jiangsu Nanjing Institute of Geo-Engineering Investigation, Nanjing 210041, China; xuch966@163.com (C.X.); tangguoyi1970@163.com (G.T.)

**Keywords:** copper slag, biochar, geopolymer composites, CO_2_ mineralization, sustainable construction materials

## Abstract

The escalating accumulation of industrial solid wastes (e.g., copper slag: CS, ground-granulated blast furnace slag: GGBS) and carbon-intensive cement production has intensified environmental challenges, driving the demand for sustainable construction materials that synergize waste valorization with carbon sequestration. This study investigates the evaluation of the compressive strength, mineralogical evolution, and real-time CO_2_ capture of the alkali-activated geopolymer composite materials by optimizing the mixed design of precursor materials (CS/GGBS ratio: 7/3) with MgO (0–10%) and coconut shell (CSB), peanut shell (PSB), and durian shell biochar (DSB) (0–3%). Results reveal that the 5% MgO addition achieves an 89.5% early-age compressive strength increase versus the MgO-free specimen. The compressive strength of the geopolymer composite could be further increased by a 1.5% dosage of DSB with an average pore size of 8.98 nm. In addition, the incorporation of an appropriate amount of porous biochar could not only enhance the CO_2_ capture capacity of the geopolymer composite, but also further improve the CO_2_ mineralization efficiency. The optimal formulation (5% MgO + 1.5% DSB) could mineralize 40.2 kg CO_2_ per ton of solid waste at least. This work highlights a sustainable strategy for synchronizing industrial solid waste valorization with carbon-negative construction providing scalable CO_2_ sequestration solutions.

## 1. Introduction

The release of greenhouse gases, particularly carbon dioxide (CO_2_), and its subsequent accumulation in the atmosphere, have undergone a pronounced escalation in recent decades [1]. The average global atmospheric concentration of CO_2_ is currently at 407 parts per million (ppm), representing the highest level recorded for the past 800,000 years [2]. In consideration of the elevated CO_2_ emissions inherent to cement production, there has been a focus on the utilization of industrial solid waste (e.g., copper slag [3,4], blast furnace slag [5,6]) and biochar (e.g., wood shavings [7], saw dust, nut shell, soft peel [8]) in research on the development of geopolymers for the purpose of CO_2_ capture. According to official data from the China Nonferrous Metals Industry Association, China produced 13.64 million tons of refined copper in 2024, generating approximately 2.2 tons of copper slag (CS) per ton of refined copper. This resulted in an annual CS output of 30 million tons in 2024, with historical stockpiles surpassing 300 million metric tons. If left unutilized, these accumulations will exacerbate land resource competition and environmental risks [9,10].

The research on the comprehensive utilization of CS is mostly focused on its application as a cementitious material [10,11,12] and construction aggregate [13,14,15]. The main phases of CS include the fayalite, magnetite, and amorphous phases [16]. As a result of their chemical composition, CS powders exhibit pozzolanic properties and are utilized as supplementary cementitious materials [17]. The best amount of CS as a supplement to cementitious materials is 5% to 15% [18,19,20]. The optimal amount of CS to replace natural sand as aggregate can reach about 60% to 80% [13,21]. Under alkaline activation conditions, ground-granulated blast furnace slag (GGBS) is usually used as a supplementary cementitious material, while CS is activated to exhibit reactivity and also functions as fine aggregate [20,22]. Manjarrez et al. [23] accelerated the geopolymer reaction of copper slag-based geopolymer binder by increasing the curing temperature (45–75 °C) to enhance the molding strength of the material. The results indicated that 60 °C might be the appropriate temperature. The process of geopolymerization involves the dissolution of solid aluminosilicates in an alkaline solution, the formation of silica–alumina oligomers, and the polycondensation of the oligomeric species forming the inorganic polymeric material [23]. Geopolymer consists of a polymerized network of [SiO_4_]^4−^ and [AlO_4_]^5−^ tetrahedra, with alkali cations such as Na^+^ or K^+^ residing in the interstices of the network for charge balance [23]. The syntheses of geopolymer composites have attracted considerable attention due to their low cost, low carbon footprint, and chemical and physical properties [24,25,26]. Therefore, the combination of CS and GGBS as an alkaline-activated full-solid-waste geopolymer mortar would be a feasible utilization method.

Furthermore, the mechanical properties and CO_2_ capture capacity of solid-waste-based polymer materials can be enhanced by incorporating magnesium oxide (MgO) and biochar [27,28]. The potential application of active MgO as a substitute for binders has the potential to reduce CO_2_ emissions [29]. The capacity of these binders to absorb CO_2_ in the form of stable carbonates through mineralization during the curing process can contribute to strength as well [30]. Specifically, magnesium-rich minerals (e.g., olivine) serve as effective additives, supplying both amorphous silica and MgO to the geopolymer system while enhancing CO_2_ mineralization through the formation of magnesium carbonates [31,32]. Furthermore, the incorporation of active MgO can enhance early strength by rapidly generating a hydrated-layer-like phase [33], which refines the pore size of pastes and increases the compressive strength [34]. However, this might also cause cracking problems for the geopolymer during the curing process [33]. Therefore, it would be appropriate to incorporate porous biochar with water-retaining properties.

The incorporation of an optimal amount of biochar enhances cement hydration by regulating moisture availability, and after carbonation, these hydrates improve bonding strength while carbonates densify the microstructure, significantly boosting both mechanical performance and CO_2_ sequestration [35]. Wang et al. [36] found that porous biochar with CO_2_ gasification could further promote the diffusion and carbonation of CO_2_. Liu et al. [37] evaluated the active carbon capture performance of artificial lightweight cold-bonded aggregates doped with biochar. The results showed that the carbon absorption of artificial lightweight cold-bonded aggregates increased with the increase in the dosage of biochar, while the compressive strength reached its peak at a 5% biochar content. Yan et al. [38] also combined modified biochar with multiple types of solid waste to develop cold-bonded artificial lightweight aggregates with high carbon sequestration capabilities and a negative carbon footprint. In the above studies on CO_2_ mineralization capture, much attention has been paid to the carbon sequestration performance of recycled aggregates mixed with biochar. Furthermore, Mhasde et al. [39] reviewed a recent trend in carbon capture with geopolymer matrix composites (GMCs), revealing the limitations of highly efficient solid adsorbents (zeolite composite adsorbents, carbon composite adsorbents, metal–organic frameworks), and explaining the advantages of GMCs, demonstrated through CO_2_ adsorption/desorption measurements, including environmental friendliness, high economic efficiency, thermal stability, and the highest CO_2_ adsorption capacity (6.09 mmol/g) at 298 K and 1 bar. But there is a lack of research on the performance of solid-waste-based geopolymer composite materials in actively absorbing CO_2_ directly from the air. In addition, there exists a paucity of studies investigating the synergistic effects of the incorporation of low-content MgO and biochar on CO_2_ mineralization and mechanical strength in CS-GGBS geopolymer.

In this study, CS and GGBS were utilized as raw materials for the preparation of alkali-activated geopolymer composite materials, while MgO and biochar served as admixtures to enhance compressive strength and carbon absorption performance. In order to obtain active silicon components from only solid waste raw materials under alkaline excitation conditions, sodium hydroxide (NaOH) was used as a single activator. More specifically, this work investigates the evaluation of the compressive strength, mineralogical evolution, and real-time CO_2_ capture of the alkali-activated geopolymer composite materials by optimizing the mixed design of precursor materials (CS/GGBS ratio: 7/3) with MgO (0–10%) and coconut shell (CSB), peanut shell (PSB), and durian shell biochar (DSB) (0–3%). This study highlights a sustainable strategy for synchronizing industrial solid waste valorization with carbon-negative construction, providing scalable CO_2_ sequestration solutions. Moreover, the engineering background of geopolymer composites in this work is not restricted. It can be a brick, mortar, or bonding material, depending on subsequent targeted expansion research. However, this does not affect the conclusions drawn from the current test data at the mechanism level regarding the compressive strength test and carbon dioxide absorption performance.

## 2. Materials and Methods

### 2.1. Materials

**Figure 1:**
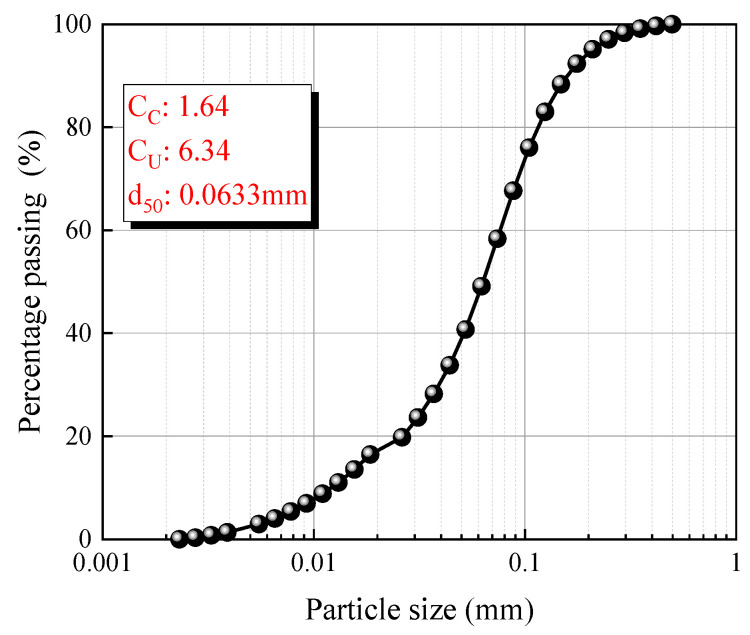
Particle size distributions of CS (Cu means Uniformity coefficient and C_C_ means Coefficient of curvature; Cu = d60/d10, Cc = (d30)^2^/(d60 × d10).

**Figure 2:**
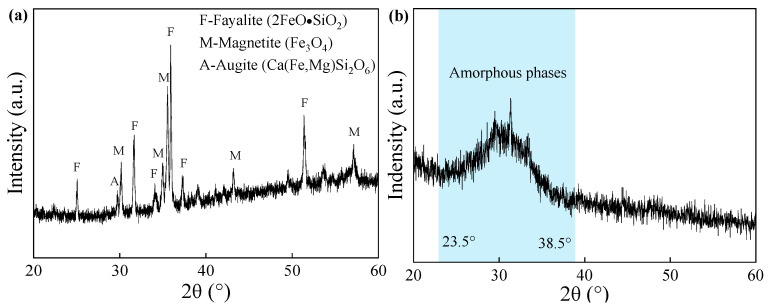
XRD patterns of raw materials: (**a**) CS, (**b**) GGBS.

The CS was obtained from Weifang, Shandong Province, China. Figure 1 shows the particle size distribution of the CS. Coefficient of curvature (C_C_) and uniformity coefficient (C_u_) were 1.64 and 6.34, respectively. The particle size of GGBS as a cementing material was less than 0.074 mm. The chemical composition of the raw materials (CS and GGBS) measured by X-ray fluorescence are listed in Table 1. The total sum of chemical composition (SiO_2_, Al_2_O_3_ and Fe_2_O_3_) exceeded the minimum requirement of 70% for natural pozzolans categorized as Class N in accordance with the ASTM C618 [40]. As a composition of C-A-S-H gel, calcium plays an important role in the reaction of geopolymer. Analyses of the mineral composition and phases of CS and GGBS were carried out through XRD analysis, as shown in Figure 2. The mineralogical composition of CS predominantly comprised iron-bearing minerals. GGBS exhibited a broad spectrum (amorphous phases) in the range of approximately 23.5–38.5°, indicating enhanced reactivity compared to crystalline phases [3]. In addition, commercial grade SH flakes (98% purity) were used as alkali activator, and MgO with a purity of 96% was used as additive.

**Figure 3:**
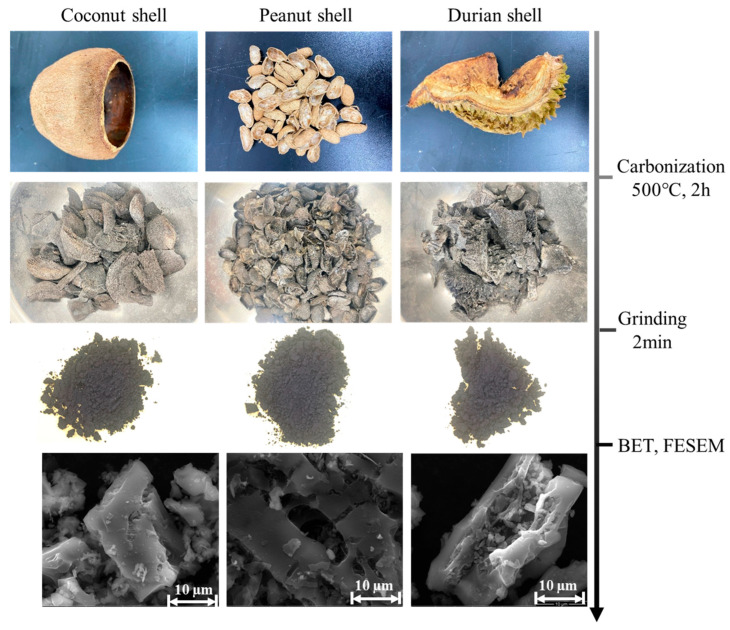
The preparation and characterization of biochar.

Peanut shells, coconut shells, and durian shells were identified as biochar raw materials, which were carbonized at 500 °C for 2 h and then mechanically granulated. The granulated biochar particles were sieved, and the portion smaller than the maximum particle size of CS (0.5 mm) was selected. Figure 3 shows the preparation and characterization of three types of biochar. Furthermore, the microscopic structure diagram illustrated in Figure 3 demonstrates that DSB had a reduced number of cracks but larger pores. The specific surface areas of PSB, CSB, and DSB were 108.54, 96.96, and 6.62 m^2^/g, respectively, and the average pore sizes were 1.99, 2.17, and 8.98 nm, respectively, as quantified by the Brunauer–Emmett–Teller (BET) method (Table 2). DSB exhibited the smallest specific surface area and the largest pore diameter. It can be observed that a reduction in pore size correlates with an increase in specific surface area. In addition, the bulk densities of CS, GGBS, CSB, MgO PSB, and DSB are 1.514, 0.813, 0.358, 0.338, 0.217, and 0.286 g/cm^2^, respectively.

### 2.2. Mixing Design and Procedure

The adopted mixture proportion comprised the material mixing ratio and curing method (Table 3). A single-factor analysis method was employed to design the material mixing proportions. CS and GGBS were used as solid-waste-based raw materials. To enhance the utilization rate of CS, its proportion was gradually increased from 60% to 100%, while the proportion of GGBS was correspondingly reduced from 40% to 0%. The dosage of MgO was set at 0–10% of the total mass of the solid waste materials. Similarly, three types of biochar were added each at 0–3% of the total weight of solid waste raw materials. Furthermore, NaOH was used as the single activator and prepared as a 12 M solution. The water required for preparing the NaOH solution accounted for 25% of the total weight of the solid waste materials. As an illustration, preliminary experiments were conducted to assess the effect of varying alkali excitation concentrations (6 M, 8 M, 10 M, 12 M) on the compressive strength of the specimens. The average compressive strengths recorded were 16.52, 19.68, 20.47, and 21.63 MPa, respectively. In order to optimize the reactivity of the solid waste raw material, a 12 M concentration NaOH solution was selected.

CS and GGBS were mixed in a certain proportion for 5 min in the laboratory concrete mixer at an rpm of 22–24 rpm. The MgO was also thoroughly mixed at this stage, and finally the biochar was added for a further period of stirring. The room temperature solution of sodium hydroxide was added in the mixer and rotated for 3 min to obtain a homogeneous mixture [21]. The mixed slurry was poured into the mold (a cylindrical mold with 50 mm diameter and 100 mm height), vibrated by a vibration table and covered by a polyethylene sheet to prevent water loss. All specimens with molds were cured at 60 °C and demolded after 24 h. All specimens were cured in an oven at 60 °C until tested. CGM-1.5D refers to the addition of DSB, which accounted for 1.5% by weight of the solid waste raw material in CG-5M.

**Figure 4:**
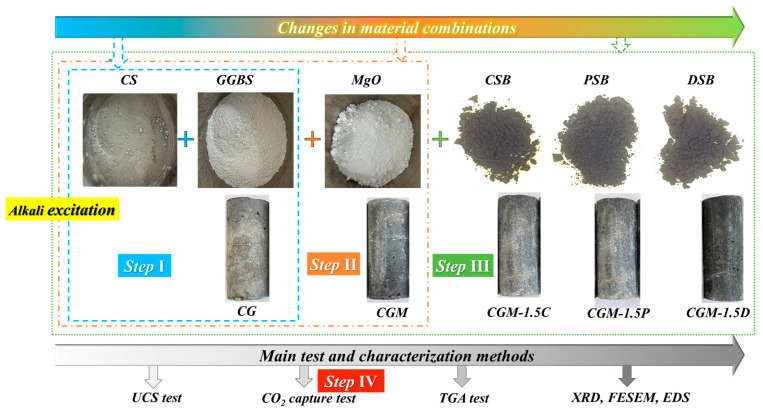
An overall roadmap of the research steps.

The whole process of testing the mixing proportion of specimens was divided into four steps (Figure 4): Step I: The optimal ratio of CS and GGBS was selected through the compressive strength test. The optimal MIX ID is CG. Step II: The optimal dosage of MgO in the CG specimen was determined through compressive strength tests. The optimal MIX ID is CGM. Step III: The optimal dosage of biochar (CSB, PSB, DSB) in CGM specimen was determined through compressive strength tests. Step IV: Based on the above steps, the CO_2_ capture of the specimen was monitored in real time. Optimization of the material mixing proportion in the first three steps was primarily accomplished by compressive strength analysis. And the final proportion of the specimen was determined after comprehensive comparison of compressive strength and CO_2_ mineralization characteristics. Five specimens (CG, CGM, CGM-1.5C, CGM-1.5P, CGM-1.5D) required for real-time CO_2_ capture monitoring after 28 days of curing are also shown in Figure 4.

### 2.3. Test Methods

A compressive strength test was carried out at 3, 7, and 28 days to determine the changes in strength of specimens with different material ratios by using a Microcomputer-controlled electronic universal testing machine (Instrument model: UTM5105, Manufacturer: Shenzhen Sansi Zongheng Technology Co., Ltd., Shenzhen, China). Specimens were taken from the curing room, covered with towels, and tested as quickly as possible to prevent water exchange with the environment. Each specimen was subjected to the compressive strength test at a constant strain rate of 1 mm/min until the load values decreased with increasing strain, and then each peak compressive strength value was recorded [41]. Each mixture was repeatedly tested three times to obtain reliable data from the compressive strength test. The crushed specimens were selected to be soaked in ethyl alcohol for 2 days and then dried at 60 °C for 24 h for characterization tests.

After curing for 28 days, the dry specimens were used for mineral phase studies using an X-ray diffractometer (XRD, Instrument model: D/max2200, Manufacturer: Rigaku Corporation, Osaka, Japan) under the following conditions: Cu Kα = 1.54178 Å; scan speed = 5°/min; scan range 2θ = 20–60°. The dry specimens were tiled on the specimen stage and introduced into the vacuum system to test the chemical composition. The specific mineral phases of the XRD peaks were referred to the PDF database in the Jade 6 software. Due to the complex mineral composition, the types and positions of the mineral phases were manually assigned to the unmodified original data map. The micromorphological analysis of the specimens was carried out using Field Emission Scanning Electron Microscopy (FESEM, Instrument model: Talos F200X G2, Manufacturer: Thermo Fisher Scientific Brno s.r.o., Waltham, MA, USA). Prior to FESEM and EDS analysis, the dehydrated specimens were cut into slices of 1–2 mm thickness and coated with gold.

The thermogravimetric analysis (TGA) of the dry specimens was documented using a thermal gravimetric analyzer (Instrument model: NETZSCH TG209 F3, Manufacturer: Netzsh Scientific Instruments Trading (Shanghai) Co., Ltd., Shanghai, China). Approximately 10 mg of the powder was weighed and placed in a specimen pan, followed by heating from an initial ambient temperature up to 1000 °C under a nitrogen flux of 100.0 mL/min. The heating rate was maintained at 10 °C /min and the weight loss was recorded using a microbalance [42,43].

**Figure 5:**
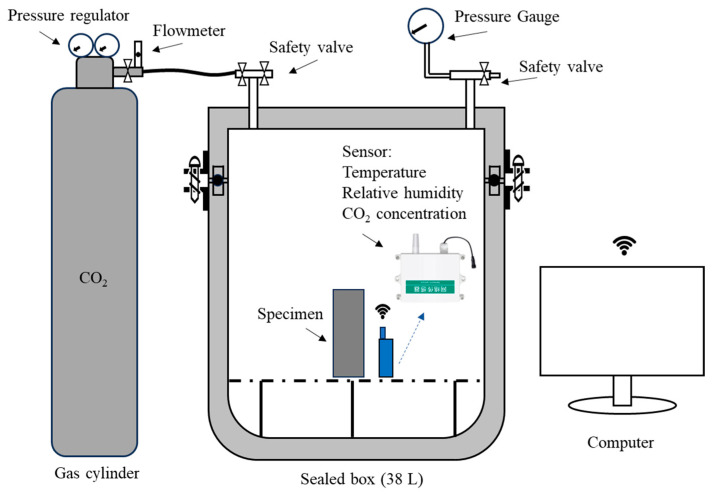
Schematic diagram of the real-time monitoring device for CO_2_ capture.

The device shown in Figure 5 was used to monitor the amount of CO_2_ captured in real time. After the quantitative CO_2_ was introduced, the sensor was used to monitor the changes in temperature, humidity, and CO_2_ concentration in the airtight container in real time to determine the CO_2_ capture characteristics of the specimen. The data was processed and stored every 2 min by the cloud platform via 4G signals. The measurable range of the 4G-sensor is 400–5000 ppm of CO_2_ concentration (±45 ppm), −10–+80 °C temperature (±0.5 °C), and 0–100% relative humidity (±3% RH). Before the test, the initial temperature, humidity, and CO_2_ concentrations in all the airtight containers were the same as those in the external environment to maintain the consistency of the initial environmental conditions for all tests. In addition, the related reactions involved in CO_2_ mineralization were summarized as follows [1,25,42,44]:

(1) $${\mathrm{CO}}_{2(g)}+H_{2}O_{\left(l\right)}\to H_{2}{\mathrm{CO}}_{3(l)}\to H^{+}+{\mathrm{HCO}}_{3}^{-}\to 2H^{+}+{\mathrm{CO}}_{3}^{2-}$$

(2) $${\mathrm{Ca}}^{2+}+{\mathrm{CO}}_{3}^{2-}\to {\mathrm{CaCO}}_{3(s)}$$

(3) $$2{\mathrm{HCO}}_{3}^{-}+{\mathrm{Ca}}^{2+}\to {\mathrm{CaCO}}_{3(s)}+{\mathrm{CO}}_{2(g)}+H_{2}O_{\left(l\right)}$$

(4) $$C-S-H_{y(s)}+H_{2}{\mathrm{CO}}_{3(l)}\to {\mathrm{CaCO}}_{3(s)}+{\mathrm{SiO}}_{2y}*H_{2}O_{\left(s\right)}$$

(5) $$2{\left(3\mathrm{CaO}*{\mathrm{SiO}}_{2}\right)}_{\left(s\right)}+3{\mathrm{CO}}_{2(g)}+3H_{2}O_{\left(l\right)}\to 3\mathrm{CaO}*2{\mathrm{SiO}}_{2}*3H_{2}O_{\left(g\right)}+3{\mathrm{CaCO}}_{3(g)}$$

(6) $$2{\left(2\mathrm{CaO}*{\mathrm{SiO}}_{2}\right)}_{\left(s\right)}+{\mathrm{CO}}_{2(g)}+3H_{2}O_{\left(l\right)}\to 3\mathrm{CaO}*2{\mathrm{SiO}}_{2}*3H_{2}O_{\left(s\right)}+{\mathrm{CaCO}}_{3(s)}$$

(7) $${\left(\mathrm{Ca},\mathrm{Mg}\right)}_{x}{\mathrm{Si}}_{y}O_{3x+2y+z}H_{2z(s)}+x{\mathrm{CO}}_{2(g)}\to x(\mathrm{Ca},\mathrm{Mg}){\mathrm{CO}}_{3(s)}+y{\mathrm{SiO}}_{2(s)}+zH_{2}O_{\left(l\right)}$$

## 3. Results and Discussion

### 3.1. Compressive Strength Analysis

**Figure 6:**
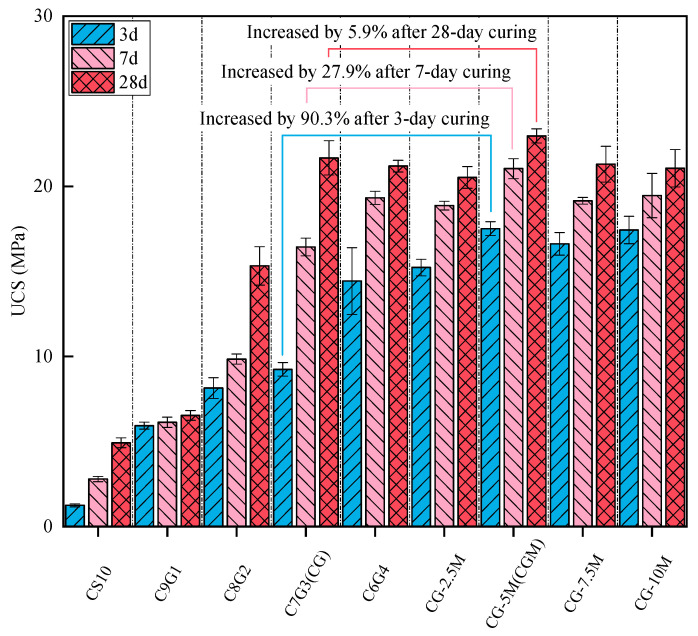
Compressive strength development with curing time for different CS-GGBS compositions and MgO dosage effects in optimal CS-GGBS mixture.

The compressive strength after 3, 7, and 28 days of curing with different proportions of CS and GGBS is shown in Figure 6. When only CS was added, the average compressive strength of the specimen at 3 d was only 1.2 MPa, but it reached about 5 MPa at 28 d. In an alkaline environment, the reactivity of CS was gradually activated, resulting in the formation of reaction products that densified the specimen structure and thereby increased the compressive strength [25]. The CS10 exhibited specific reactivity, but not enough to be used as a building material. This deficiency may be attributable to the relatively high proportion of crystalline phases in CS, which could impede dissolution and limit the production of alkali activation products [45]. The added GGBS supplemented the CaO, SiO_2_, and Al_2_O_3_ contents of the specimen. The main phases of GGBS were amorphous, which facilitated their dissolution in alkali solutions. Geopolymer gels were produced by the rapid reaction of all mixed materials [46]. The additional geopolymer reaction products could enhance the strength at early ages [47,48]. With the increasing proportion of GGBS (0–40%), the growth of the average compressive strength over 28 d initially increased before subsequently decreasing, and the peak value was 21.2 MPa (C7G3). Consequently, the optimal mixture ratio specimen was identified as C7G3 (further simplified as CG). As solid waste raw materials, the optimal content of CS and GGBS was determined to be 70% and 30%, respectively. It could be observed that CS exhibited a certain reactivity and could be utilized as a cementing material. When the highly active material GGBS was added, the unreacted CS functioned as a fine aggregate. XRD results (discussed in a subsequent section) also demonstrated that in the geopolymer mortar specimens, the presence of the CS unreacted phase acted as an inactive particle filler [49].

When 2.5%, 5%, 7.5%, and 10% MgO were added to CG, the early compressive strength increased by 65.6–89.5%. However, the compressive strength of the specimen exhibited a trend of first rising and then decreasing. This phenomenon is consistent with the test results of [50,51]. The early compressive strength of the specimen was enhanced due to the presence of MgO, which accelerated the geopolymer reaction process and produced a greater number of geopolymer reaction products, resulting in high compressive strength in the early stage [33]. Under the condition of high MgO content, a greater Hydrotalcite-like phase would be generated, which would expand and increase the volume, resulting in an unstable volume for the specimen, thus reducing the compressive strength of the specimen [52]. The optimal dosage of MgO was determined to be 5%.

**Figure 7:**
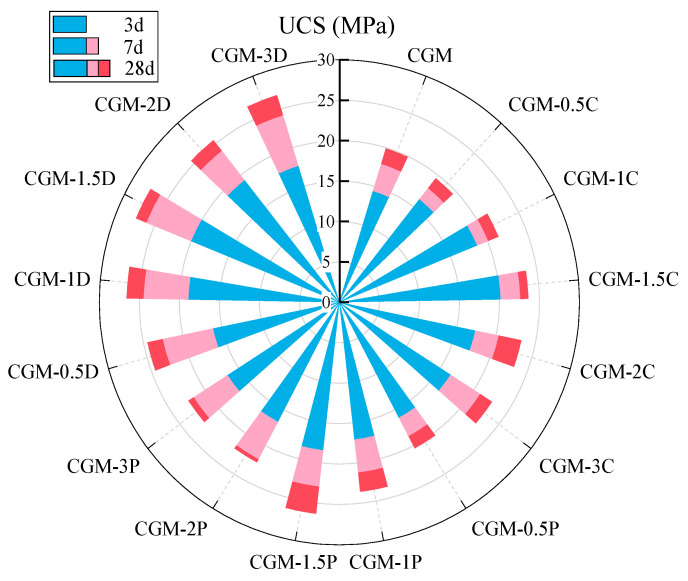
Influence of biochar type (CSB, PSB, DSB) and dosage (0.5%, 1.0%, 1.5%, 2.0%, 3.0%) on the strength of CGM specimens (CGM denotes the specimen composed of solid waste raw materials (CS/GGBS, 7/3) with 5% MgO, while C, P, and D represent coconut shell, peanut shell, and durian shell biochar, respectively).

Figure 7 shows the compressive strength development of CG-5M (further simplified as CGM) specimens incorporating different biochar types (CSB, PSB, DSB) at varying dosages (0.5–3.0%) during curing (3–28 days). Given the substantial number of specimens displayed in the figure, and to facilitate comparison with the summary specification, the data presented are the average compressive strengths. Adding 1.5% biochar content (CGM-1.5C, CGM-1.5P, CGM-1.5D) showed better compressive strength in each type of biochar-added specimen at different curing ages, which was higher than the compressive strength of CGM without biochar. The optimal ratio of each biochar was determined to be 1.5%. The addition of biochar increased the strength of the specimen due to its relatively high specific surface area, which promotes the formation of geopolymer gels through the nucleation effect [36,53]. However, an excessive dosage of biochar would reduce the compressive strength of the specimen due to its porous and brittle structure [35]. As shown in Table 2, the average pore sizes of CSB and PSB were found to be around 1.99 and 2.17 nm, respectively. DSB was determined to be around 8.98 nm. Despite the lower specific surface area of DSB, the specimen with 1.5% DSB content (CGM-1.5D) exhibited the highest average compressive strength among all types of biochar across all curing ages, at 20.0 MPa (3 d), 26.1 MPa (7 d), and 27.5 MPa (28 d), respectively. In comparison with CGM, the average compressive strength of CGM-1.5D increased by 16.9% (3 d), 29.8% (7 d), and 29.5% (28 d). These findings suggest that the presence of biochar with an 8.98 nm pore size is more conducive to enhancing the strength of the specimen.

Furthermore, the average compressive strength of CG, CGM, CGM-1.5C, CGM-1.5P, and CGM-1.5D specimens after 28 days of curing were 21.2 MPa, 22.9 MPa, 23.6 MPa, 26.3 MPa, and 27.4 MPa, respectively. The compressive strength of the specimens all met the requirements of the relevant specifications summarized in Table 4, and the satisfactory performance provided guidance for the comprehensive utilization of CS and GGBS.

### 3.2. Analysis of CO2 Real-Time Monitoring

**Figure 8:**
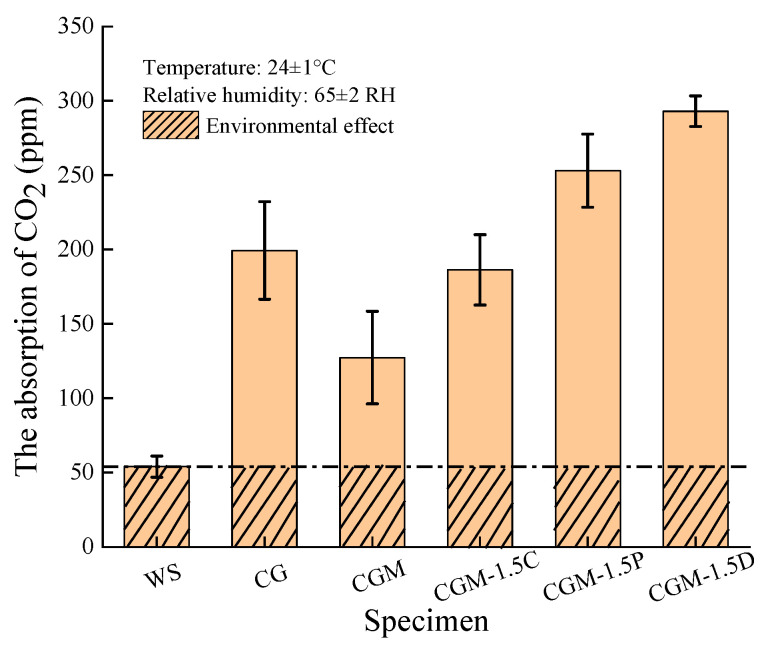
The 24 h real-time monitoring CO_2_ capture of specimens after 28 days of curing.

Five specimens (CG, CGM, CGM-1.5C, CGM-1.5P and CGM-1.5D), which had been cured for 28 days, were placed separately in a 38 L airtight container. 120 ml of CO_2_ was then injected into the container and the amount captured by the specimens was measured over 24 hours (Figure 8). To avoid the effects of moisture in the air and monitoring devices, a closed chamber without specimen (WS) was created. CGM showed lower CO_2_ capture performance compared to CG. This may be because MgO enhances the reactivity of the geopolymer reaction, resulting in increased densification of the specimen. Adding porous biochar (CSB, PSB and DSB) improved the CO_2_ capture performance of the specimens.

Compared with CGM, the CO_2_ capture of CGM-1.5C, CGM-1.5P, and CGM-1.5D increased by 78%, 179%, and 234% (Table 5), respectively. In addition, the CO_2_ capture of CGM-1.5D was 1.75 times (0.06318 mg g^−1^/0.03613 mg g^−1^) that of CG. This may be due to the addition of porous biochar, which improved the specimen structure. Also, more geopolymer reaction products were involved in the mineralization reaction. A more detailed presentation of the data and analysis is demonstrated in the thermogravimetric analysis. The addition of an appropriate amount of biochar effectively improved the mechanical properties and CO_2_ capture of specimens. The optimal dosages of CS and GGBS were 70% and 30%, and the optimal dosages of MgO and DSB were 5% and 1.5% of solid waste raw material (CS + GGBS), respectively.

**Figure 9:**
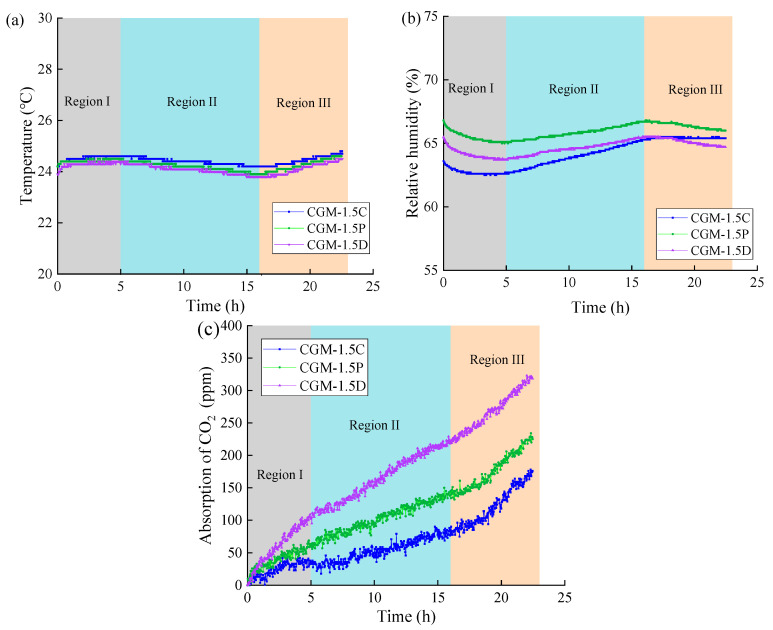
The changes in (**a**) temperature, (**b**) relative humidity, and (**c**) CO_2_ concentration during the real-time monitoring.

After 28 days of curing, the changes in temperature, relative humidity, and CO_2_ concentration within 24 h in the airtight container containing the three specimens (CGM-1.5C, CGM-1.5P, and CGM-1.5D) are shown separately in Figure 9. The data was divided into three regions (I, II, III) based on temperature variations. In regions I and III, the CO_2_ concentration increased rapidly, while the relative humidity decreased, and the temperature increased. An increase in temperature could lead to an acceleration in the rate of molecular thermal motion, which is advantageous for the capture of CO_2_. An increase in temperature could lead to an increase in saturated humidity, which in turn could lead to a decrease in relative humidity. In addition, it can also be observed that the CO_2_ capture rate of CGM-1.5D was faster than that of CGM-1.5C and CGM-1.5P in each monitoring stage, which reflects the excellent CO_2_ capture performance of CGM-1.5D. Furthermore, it was noted that the CO_2_ capture rate of CGM-1.5D was consistently higher than that of CGM-1.5C and CGM-1.5P across all monitoring stages, indicating the superior CO_2_ capture capabilities of CGM-1.5D.

### 3.3. TGA

**Figure 10:**
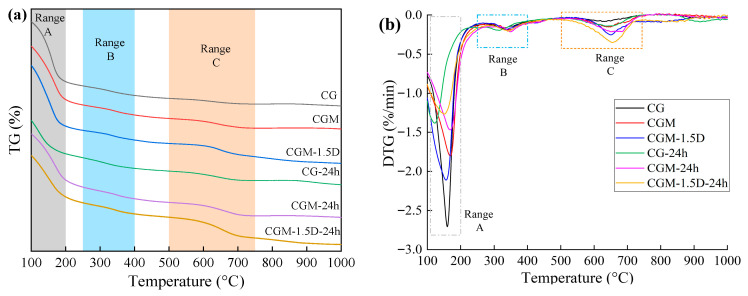
TG (**a**) and DTG (**b**) curves of three specimens (CG, CGM, CGM-1.5D).

Thermogravimetry tests were carried out on three specimens (CG, CGM, CGM-1.5D) before and after the 24 h real-time monitoring test to determine the CO_2_ mineralization properties of the specimens (Figure 10). Furthermore, the weight variations in CG, CGM, and CGM-1.5D over the three main temperature ranges (105–200 °C, 250–400 °C, and 500–750 °C) are summarized in Table 6. The decomposition peak of temperature range A (105–200 °C) was mainly due to the dehydration of micropores and the decomposition of aluminosilicate hydrates and silicate gel [35,53], The decomposition peak of temperature range B (250–400 °C) was mainly due to the decomposition of hydroxides, which contribute to the mineralization of CO_2_ [60,61,62]. The decomposition peak of temperature range C (500–750 °C) was confirmed to be caused by the decomposition of carbonate [61,62]. After 24 h monitoring, the proportion of bound water decreased significantly, while the proportion of hydroxides increased slightly. This may be due to the fact that the bound water in the specimen participated in the CO_2_ mineralization reaction during the 24 h monitoring process while the geopolymer reaction was also in progress. In the process of specimen preparation (which includes the material mixing and curing stages), the amount of CO_2_ mineralization by CGM was 1.73 times (2.01%) that of CG (1.17%). These results indicate that incorporating MgO into the CG significantly enhanced its capacity to mineralize CO_2_ during the preparation stage. In the real-time monitoring stage, the CO_2_ capture amount of the CGM-24h was only 0.52 times (0.01894 mg g^−1^/0.03613 mg g^−1^) that of the CG-24h, but the CO_2_ mineralization amount was 0.94 times (0.75%/0.80%). Although the MgO-added specimen exhibited a diminished CO_2_ capture capacity during the monitoring stage, it achieved more efficient CO_2_ mineralization. In addition, the porous characteristic of 1.5% DSB improved the formation of hydration products and the structure of CGM, and the amount of CO_2_ mineralization during specimen preparation was further increased by 2.34 times (2.74%). In the course of the 24 h monitoring stage, the amount of mineralization of CO_2_ by the CGM-1.5D-24h was 2.31 times (1.85%/0.80%) higher than that by CG-24h. It was significantly higher than 1.75 times (0.06318 mg g^−1^/0.03613 mg g^−1^) the amount of CO_2_ captured. It could be concluded that the incorporation of an appropriate amount of biochar not only significantly increases the capture amount of CO_2_, but also further improves the mineralization efficiency of CO_2_.

To further summarize, the addition of MgO could promote the mineralization of CO_2_ by CS and GGBS during the specimen preparation processes. The addition of DSB not only enhanced the geopolymer reaction, but also significantly improved the CO_2_ mineralization capacity of the cured specimen. Based on the weight proportion data of CG-, CGM-, and CGM-1.5D-mineralized CO_2_, each ton of solid waste materials (CS/GGBS, 7/3) could mineralize 18.4 kg, 25.9 kg, and 40.2 kg of CO_2_. Since CGM-1.5D exhibited the highest CO_2_ mineralization during both specimen preparation and the 24 h monitoring period, it was identified as the specimen with the optimal material ratio.

### 3.4. XRD Analysis

**Figure 11:**
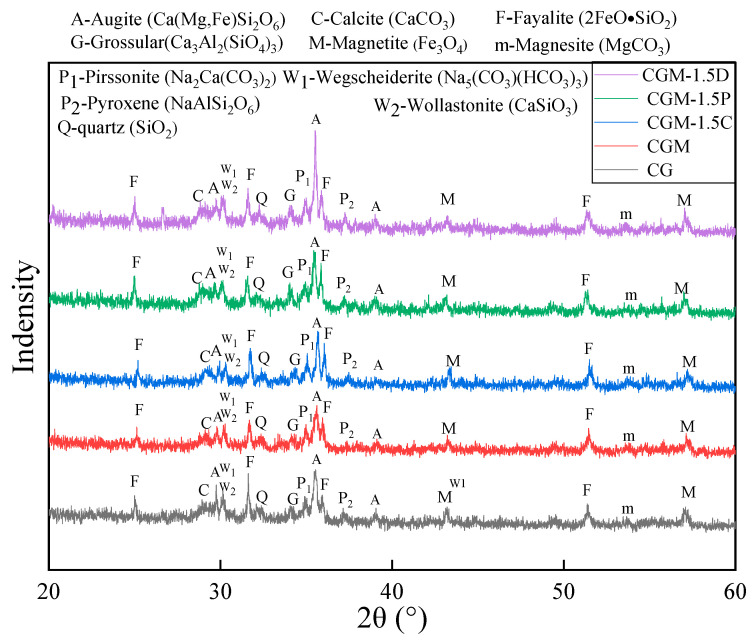
XRD curves of five specimens (CG, CGM, CGM-1.5C, CGM-1.5P, CGM-1.5D).

The five steps of specimens (CG, CGM, CGM-1.5C, CGM-1.5P, CGM-1.5D) of CS and GGBS under alkali excitation generated a large number of co-existing silicate substances (Figure 11), which provided the necessary strength for the specimens [46]. The presence of magnetite was detected as one of the compositions of CS. It could be inferred that CS not only functioned as a cementing material but also contributed to the development of specimen strength as an aggregate. Compared with the XRD curves of CS (Figure 2a), the new mineral phase of the five specimens identified silicate minerals such as augite, grossular, and wollastonite in a broad and diffuse hump (around 30 and 35°), usually indicating the presence of amorphous phases [63]. As observed, these amorphous phases are related to the formation of aluminosilicate gels [64,65]. The peak intensity of magnesium silicate minerals increased with the addition of MgO. The peaks corresponding to silicates and aluminosilicates were further enhanced after the incorporation of biochar. Combined with the compressive strength analysis, it can be seen that the addition of MgO enhanced the early strength of the specimens through the formation of magnesium silicate minerals, while biochar contributed to long-term strength development by continuously promoting the geopolymer products (augite, grossular). In addition, carbonates were also detected by XRD, which might be the products obtained from the mineralization reaction of the specimen with CO_2_. Compared with the CG without MgO, the addition of MgO increased the peak of MgCO_3_. Subsequent to the addition of biochar (CSB, PSB, DSB), especially DSB, a further elevation in the peaks of carbonate was observed. Concurrently, the peaks within the 30 to 31° range demonstrated the presence of SiO_2_ in the specimen, which in addition to the amount of SiO_2_ contained in the solid waste material (Table 3), may also be due to the mineralization reaction of CO_2_ (Equations (1)–(7)).

### 3.5. Microstructural Analysis

**Figure 12:**
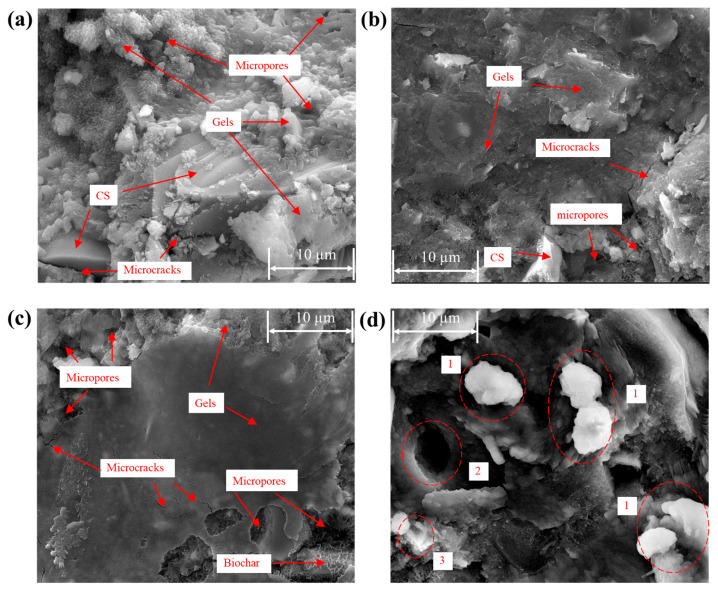
Microstructure of three specimens after 28 days of curing: (**a**) CG, (**b**) CGM, and (**c**) CGM-1.5D, (**d**) CGM-1.5D-24h.

The SEM micrographs of the three specimens (CG, CGM, CGM-1.5D) after 28 days of curing and the specimen (CGM-1.5D-24h) after real time monitoring of CO_2_ capture are shown in Figure 12. The principal binding phase exhibited a dense morphology, signifying the presence of C-S-H, C-A-S-H, and N-A-S-H gels [41]. The continuous gel held the aggregate together, while some honeycomb gel was in the hole or attached to the surface of the matrix, especially in the CG specimen. Some gels were independent particles, which were not tightly bonded with others. The aforementioned observation was consistent with the findings of Yan et al. [10] and Jin et al. [46]. In comparison with CG, both CGM and CGM-1.5D exhibited a denser microstructure, while CGM-1.5D exhibited an enlarged microporous structure in relation to CGM. Some microcracks and pores of the specimen due to the addition of biochar might have a negative effect on mechanical strength [35], but the increase in mechanical strength through adding the appropriate amount of biochar will completely offset the negative effect (combined with the analysis of compressive strength). When combined with XRD and TGA, the addition of biochar increased the formation of N-A-S-H and C-S-H, thereby improving the strength of the specimen. Unlike other specimens, the CGM-1.5D-24h specimen that underwent the CO_2_ monitoring test exhibited aggregates (red circle 1) on its surface. The pores (red circle 2) of similar size to the aggregates nearby might be caused by the shedding of the aggregates during the testing process. A substance highly similar in morphology and structure to DSB (red circle 3) was found to be encapsulated with gels. Furthermore, some aggregates could be observed in the vicinity of the pore openings.

**Figure 13:**
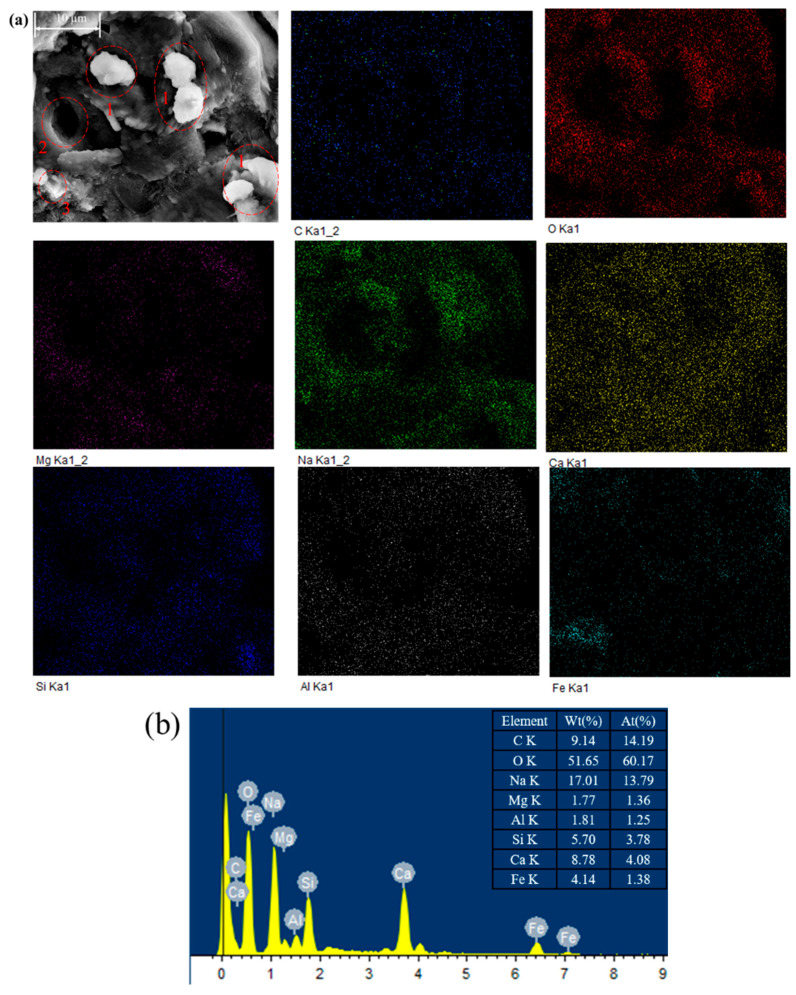
The EDS charts of CGM-1.5D-24h: (**a**) distribution of elements (C, O, Mg, Na, Ca, Si, Al, Fe), (**b**) corresponding weight percent and atomic percent.

Figure 13 shows the EDS charts of CMM-1.5D-24h. The widely and uniformly distributed O, Na, Ca, Si, and Al elements (Figure 13a) revealed that the geopolymer reaction products of CS and GGBS under the excitation of the NaOH solution were widely and continuously distributed. Additional aggregates (red circle 1) were found to contain overlapping elements of C, Ca, O, Na, Si, Al, and trace amounts of Mg. This outcome may be attributable to the concurrent occurrence of the geopolymer reaction and the CO_2_ mineralization reaction during the real-time monitoring of CO_2_ capture. Furthermore, the C element was found to widely overlap with O, Mg, Ca, and Na, and both the weight percent and atomic percent of C were within the top three ranges (Figure 13b), indicating that carbonates were widely present in the specimen and demonstrating the excellent CO_2_ mineralization performance of the specimen. It is worth noting that the proportion of sodium was abnormally higher than that of silicon. This was similar to choosing a NaOH solution with too high a concentration (12 M). To enhance the reactivity of CG and GGBS, an 8 M concentration was not selected, and it was not combined with sodium silicate.

As an illustration, the quantitative analysis of CO_2_ absorption was determined through real-time monitoring of CO_2_ concentration changes in tests and TGA. XRD, FESEM and EDS were used for qualitative analysis of the mineral phase changes, microstructure changes, and elemental distribution of specimens before and after the real-time monitoring test.

### 3.6. Discussion

**Figure 14:**
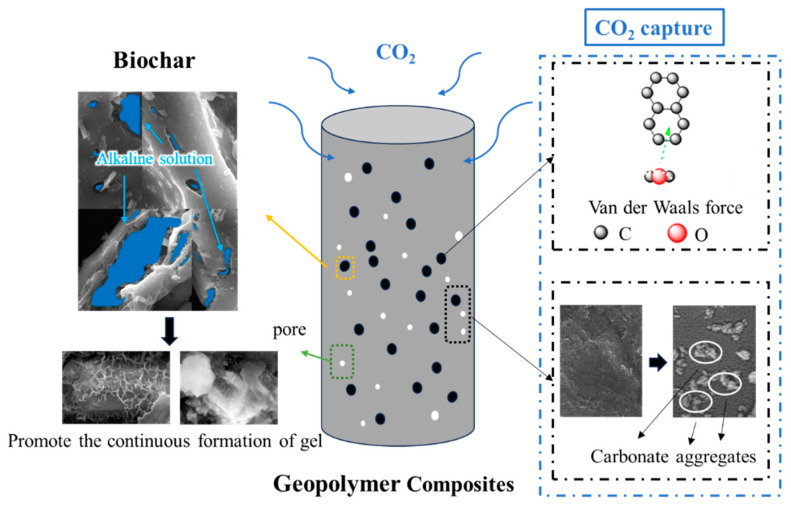
Mechanism of CO_2_ capture by geopolymer composites supplemented with biochar.

Figure 14 provides a visualization of the mechanism of the incorporation of biochar acting on the strength increase and CO_2_ capture of geopolymer composites. The addition of an appropriate amount of biochar can continuously generate geopolymer gels through the slow release of alkaline solutions to improve the mechanical strength of geopolymer composites. In contrast to the extreme test environments involving high pressure, high humidity, and full CO_2_, this study investigated the active capture of lower concentrations of CO_2_ by geopolymer composites of porous biochar under normal pressure and normal humidity conditions. This process involves physical absorption and chemical mineralization. The former, physical absorption, mainly includes van der Waals forces. The mineralization of CO_2_ involves complex chemical reactions (Equations (1)–(7)), and the products of these mineralization reactions are interwoven and distributed with the products of geopolymer reactions. The mineralization efficiency of CO_2_ is contingent on the content of metal cations, including Ca^2+^ and Mg^2+^. When metal oxides in multi-source solid waste are fully activated, it will help to comprehensively utilize solid waste while reducing the carbon footprint of composite materials. Compared with geopolymer composites without biochar, the incorporation of an appropriate amount of biochar can not only improve their mechanical strength but also enhance the performance of active carbon dioxide capture, especially the performance of the mineralization of CO_2_. If the pores of the biochar and their distribution in the geopolymer composites are intentionally designed, its positive role may be further expressed.

Predictably, when sodium silicate or activated silicon-rich mineral is added, the mechanical properties of geopolymer composites can be further enhanced. However, when sodium silicate and sodium hydroxide are used as combined activators to enhance the performance of composite materials, this might be weakened due to the fact that the products generated by the rapid reaction of GGBS with sodium silicate under alkaline activation conditions quickly coat the particles of CS, resulting in the insufficient activation of CS. From the perspective of a green economy and considering non-load-bearing application scenarios, it is of great significance to improve the utilization rate of CS and achieve the application of research on full-solid-waste materials under low-concentration or alkali-free activator conditions.

## 4. Conclusions

In this research, geopolymer composites prepared from CS and GGBS with varying dosages of MgO and biochar through alkali excitation were investigated for their mechanical properties. In addition, a series of tests were conducted to evaluate the CO_2_ capture and mineralization capabilities of geopolymer composites under normal temperature, normal pressure, and low CO_2_ concentration conditions, with a corresponding analysis of mineral phase and microstructure changes. The significant results can be summarized as follows:

(1) CS plays a role not only as a cementitious material, but also as a fine aggregate in geopolymer composites produced from solid waste materials. The addition of MgO enhances the early strength of the specimens, while biochar contributes to the long-term strength development.
(2) As well as significant improvements in early strength (89.5%), 5% dosage of MgO added into CG can also improve the mineralization performance of CO_2_ in the preparation process. Although the CGM specimen exhibited diminished CO_2_ capture capacity during the monitoring stage, it achieved more efficient CO_2_ mineralization than the CG specimen.
(3) The compressive strength of the geopolymer composite could be further increased by a 1.5% dosage of DSB through improving the pore structure and producing more gels (27.5 MPa at 28 days). In addition, the incorporation of an appropriate amount of porous biochar could not only enhance the CO_2_ capture capacity of the geopolymer composite but also improve the efficiency of CO_2_ mineralization.
(4) DSB with an average pore size of 8.98 nm is more conducive to strength growth and CO_2_ mineralization than CSB and PSB with average pore sizes of around 1.99 and 2.17 nm, respectively.
(5) The geopolymer composite formulated with solid waste materials (CS/GGBS, 7/3), 5% MgO, and 1.5% DSB represents the optimal mix ratio, demonstrating a compressive strength of 27.5 MPa, a CO_2_ mineralization weight ratio of 4.28%, and a CO_2_ capture capacity within a 24 h real-time monitoring test of 0.06318 g/kg. This optimized material formulation not only satisfies specific compressive strength requirements but also achieves a minimum CO_2_ mineralization capacity of 40.2 kg per ton of solid waste (CS/GGBS = 7/3), operating effectively without energy-intensive conditions (high temperature, pressure, humidity, or CO_2_ concentration).

## Figures and Tables

**Table 1:** Oxide composition of CS and GGBS.

Oxide Composition (wt.%)	CS	GGBS
Fe_2_O_3_	52.90	0.27
SiO_2_	25.78	27.22
Al_2_O_3_	6.05	15.86
CaO	4.60	37.74
Na_2_O	2.82	0.67
ZnO	1.94	-
MgO	1.72	7.63
K_2_O	1.16	0.34
TiO_2_	0.35	1.14
CuO	0.27	-
PbO	0.25	-
MnO	0.21	0.26
As_2_O_3_	0.19	-
P_2_O_5_	0.13	0.02
Cr_2_O_3_	0.05	-

**Table 2:** The specific surface area and average pore diameter of biochar.

Biochar	Specific Surface Area (m^2^/g)	Average Pore Size (nm)
Coconut shell biochar (CSB)	108.54	1.99
Peanut shell biochar (PSB)	96.96	2.17
Durian shell biochar (DSB)	6.62	8.98

**Table 3:** Mixing proportions of specimens (wt.%).

Step	Mix ID	The Ratio of Solid Waste Raw Materials		MgO (%)	Biochar		NaOH (M)	W (%)
		CS %	GGBS %		**(%)**	**Type**		
I	CS10	100	0	-	-	-	12.0	25.0
	C9G1	90	10	-	-	-		
	C8G2	80	20	-	-	-		
	C7G3 (CG)	70	30	-	-	-		
	C6G4	60	40	-	-	-		
II	CG-2.5M	70	30	2.5	-	-		
	CG-5M (CGM)	70	30	5.0	-	-		
	CG-7.5M	70	30	7.5	-	-		
	CG-10M	70	30	10.0	-	-		
III	CGM-0.5C	70	30	5.0	0.5	CSB		
	CGM-1C	70	30	5.0	1.0			
	CGM-1.5C	70	30	5.0	1.5			
	CGM-2C	70	30	5.0	2.0			
	CGM-3C	70	30	5.0	3.0			
	CGM-0.5P	70	30	5.0	0.5	PSB		
	CGM-1P	70	30	5.0	1.0			
	CGM-1.5P	70	30	5.0	1.5			
	CGM-2P	70	30	5.0	2.0			
	CGM-3P	70	30	5.0	3.0			
	CGM-0.5D	70	30	5.0	0.5	DSB		
	CGM-1D	70	30	5.0	1.0			
	CGM-1.5D	70	30	5.0	1.5			
	CGM-2D	70	30	5.0	2.0			
	CGM-3D	70	30	5.0	3.0			

Note: C7G3 represents the weight ratio of CS and GGBS in solid waste raw materials, which is 7/3. CG only represents the further simplification of C7G3. CG-5M represents the addition of MgO accounting for 5% by weight of the solid waste raw materials in CG. CGM only represents the further simplification of CG-5M. CGM-1.5D represents the addition of durian shell biochar (DSB), which accounted for 1.5% by weight of the solid waste raw materials in CG-5M, while C, P, and D represent coconut shell (CSB), peanut shell (PSB), and DSB, respectively. The water required for preparing thr NaOH solution accounted for 25% of the total weight of the solid waste materials.

**Table 4:** Summary of relevant specifications.

Standard Code	Strength Grade	Average Compressive Strength (MPa)	Application
GB/T 21144-2023 [54]	MU20	20.0	Use for buildings or structures
	MU15	15.0	
	MU10	10.0	
JC/T 422-2007 [55]	MU20	20.0	
	MU15	15.0	
JC/T 239-2014 [56]	MU20	20.0	Use for industrial and civil buildings
	MU15	15.0	
ASTM C216-24 [57]	SW	20.7	Use for buildings or structures
	MW	17.2	
ASTM C62-23 [58]	SW	20.7	
	MW	17.2	
	NW	10.3	
ASTM C902-22 [59]	MX	17.2	Use as paving material
	NX	17.2	

**Table 5:** The CO_2_ capture by monitoring.

Specimen	The Absorption of CO_2_ (g)	The Absorption of CO_2_ per Unit Weight (mg/g)
CG	0.0099	0.03613
CGM	0.0050	0.01894
CGM-1.5C	0.0090	0.03371
CGM-1.5P	0.0136	0.05292
CGM-1.5D	0.0163	0.06318

**Table 6:** Quantitative analysis of TG.

Specimens	TG			Proportion of CO_2_ Mineralization in 24 h Monitoring (%)
	A (105–200 °C, %)	B (250–400 °C, %)	C (500–750 °C, %)	
CG	12.39	1.97	1.17	-
CGM	11.02	2.07	2.01	-
CGM-1.5D	12.55	2.00	2.74	-
CG-24h	6.90	2.19	1.96	0.80
CGM-24h	9.48	2.41	2.76	0.75
CGM-1.5D-24h	8.56	2.29	4.28	1.85

Note: CGM-1.5D-24 refers to the CGM-1.5D that was monitored for 24 h. CGM-1.5D refers to the specimen prepared with solid waste materials (CS/GGBS, 7/3), with 5% MgO and 1.5% DSB.

## Data Availability

The original contributions presented in this study are included in the article. Further inquiries can be directed to the corresponding author.

## Abbreviations

CO_2_	carbon dioxide
CS	copper slag
GGBS	ground-granulated blast furnace slag
MgO	magnesium oxide
NaOH	sodium hydroxide
CSB	coconut shell biochar
PSB	peanut shell biochar
DSB	durian shell biochar
C_C_	Curvature coefficient
C_u_	uniformity coefficient
12 M	12 mol/L
TGA	The thermogravimetric analysis
XRD	X-ray diffractometer
FESEM	Field Emission Scanning Electron Microscopy
EDS	Energy Dispersive Spectrometer
C-S-H	Calcium Silicate Hydrate
C-A-S-H	calcium alumina silicate hydrate
N-A-S-H	sodium alumina silicate hydrate
C7G3	the weight ratio of CS and GGBS in solid waste raw materials is 7/3
CG	the further simplification of C7G3
CG-5M	the addition of MgO accounting for 5% by weight of solid waste raw materials in CG
CGM	the further simplification of CG-5M
CGM-1.5D	the addition of DSB, which accounted for 1.5% by weight of the solid waste raw materials in CG-5M
